# Confidence-based progress-driven self-generated goals for skill acquisition in developmental robots

**DOI:** 10.3389/fpsyg.2013.00833

**Published:** 2013-11-26

**Authors:** Hung Ngo, Matthew Luciw, Alexander Förster, Jürgen Schmidhuber

**Affiliations:** IDSIA, Dalle Molle Institute for Artificial Intelligence, Università della Svizzera Italiana-Scuola Universitaria Professionale della Svizzera Italiana (USI-SUPSI)Lugano, Switzerland

**Keywords:** intrinsic motivation, artificial curiosity, continual learning, developmental robotics, online active learning, markov decision processes, AI planning, systematic exploration

## Abstract

A reinforcement learning agent that autonomously explores its environment can utilize a curiosity drive to enable continual learning of skills, in the absence of any external rewards. We formulate curiosity-driven exploration, and eventual skill acquisition, as a selective sampling problem. Each environment setting provides the agent with a stream of instances. An instance is a sensory observation that, when queried, causes an outcome that the agent is trying to predict. After an instance is observed, a query condition, derived herein, tells whether its outcome is statistically known or unknown to the agent, based on the confidence interval of an online linear classifier. Upon encountering the first unknown instance, the agent “queries” the environment to observe the outcome, which is expected to improve its confidence in the corresponding predictor. If the environment is in a setting where all instances are known, the agent generates a plan of actions to reach a new setting, where an unknown instance is likely to be encountered. The desired setting is a self-generated goal, and the plan of action, essentially a program to solve a problem, is a skill. The success of the plan depends on the quality of the agent's predictors, which are improved as mentioned above. For validation, this method is applied to both a simulated and real Katana robot arm in its “blocks-world” environment. Results show that the proposed method generates sample-efficient curious exploration behavior, which exhibits developmental stages, continual learning, and skill acquisition, in an intrinsically-motivated playful agent.

## 1. Introduction

During our lifetimes, we continually learn, and our learning is often intrinsically motivated (Piaget, 1955; Berlyne, 1966). We do not just learn declarative knowledge, such as that exhibited by contestants appearing on the popular quiz show *Jeopardy*, but also procedural knowledge, such as how to write a Ph.D. thesis. In general, a skill is a program able to solve a limited set of problems (Schmidhuber, 1997; Srivastava et al., 2013), but the notion of a skill is often coupled with procedural knowledge, which is typically demonstrated through action. In continually learning artificial agents, skill acquisition (Newell et al., 1959; Ring, 1994; Barto et al., 2004; Konidaris, 2011; Lang, 2011; Sutton et al., 2011) is a process involving the *discovery* of new skills, learning to *reproduce* the skills reliably and efficiently, and *building upon* the acquired skills to support the acquisition of more skills. This process should never stop. An eventual goal of ours, and others, is the development of lifelong learning robot agents (Ring, 1994; Thrun and Mitchell, 1995; Ring, 1997; Sutton et al., 2011).

Traditional Markovian Reinforcement Learning (RL) (Sutton and Barto, 1998; Szepesvári, 2010) provides a formal framework that facilitates autonomous skill acquisition. In the Markov Decision Process (MDP) framework, a skill is represented as a policy that, when executed, is guaranteed to efficiently reach a particular state, which would be a “goal” state for that skill. RL involves optimizing a policy, to allow the agent to achieve the maximum expected reward.

There exist iterative *planning* methods, such as value iteration (Bellman, 1957) and policy iteration (Howard, 1960), to find an optimal policy for an MDP if a *model* of the environment is *known* to the agent; see (Mausam and Kolobov, 2012) for recent reviews. The model is the set of transition probabilities *P*(*s*_*t* + 1_|*s_t_, a_t_*) of reaching successor state *s*_*t* + 1_, together with the associated expected immediate rewards *R*(*s_t_, a_t_*) when the agent takes action *a*_*t*_ in state *s*_*t*_. By selecting different goal states and creating appropriate “phantom” rewards, which are not provided by the environment, the agent could calculate a policy for a self-generated goal immediately through planning (Luciw et al., 2011; Hester and Stone, 2012; Ngo et al., 2012). An autonomous skill learner for model-based Markovian RL needs only learn a single transition model (or another type of predictive world model) and to be able to generate a different reward function for each skill.

An important issue in learning a world model is *systematic exploration*. How can an agent explore the environment to quickly and effectively learn? Early methods were based on common-sense heuristics such as “visit previously unvisited states,” or “visit states that have not been visited in a while” (Sutton, 1990). More recent methods are those based on *Artificial Curiosity* (Schmidhuber, 1991; Storck et al., 1995; Wiering and Schmidhuber, 1998; Meuleau and Bourgine, 1999; Barto et al., 2004; Şimşek and Barto, 2006; Schmidhuber, 2010; Ngo et al., 2011), which can be exploited in developmental robotics (Weng et al., 2001; Lungarella et al., 2003; Oudeyer et al., 2007; Asada et al., 2009; Hester and Stone, 2012; Ngo et al., 2012).

Artificial curiosity uses an intrinsic reward, which is the *learning progress*, or expected improvement, of the adaptive world model [i.e., predictor/compressor of the agent's growing history of perceptions and actions (Schmidhuber, 2006)]. The expected learning progress becomes an intrinsic reward for the reinforcement learner. To maximize expected intrinsic reward accumulation, the reinforcement learner is motivated to create new experiences such that the adaptive learner makes quick progress.

We investigate an autonomous learning system that utilizes such a progress-based curiosity drive to explore its environment. This is a “pure exploration” setting, as there are no external rewards. The general framework is formulated as a selective sampling problem in which an agent samples any action in its current situation as soon as it sees that the effects of this action are statistically unknown. We present one possible implementation of the framework, using online linear classifiers (Azoury and Warmuth, 2001; Vovk, 2001; Cesa-Bianchi and Lugosi, 2006) as *predictive action models*, which essentially predict some aspects of the next state, given the current state-action features.

If no available actions have a statistically unknown outcome, the agent generates a plan of actions to reach a new setting where it expects to find such an action. The planning is implemented using approximate policy iteration, and depends on the procedural knowledge accumulated so far in the adaptive world model. The agent acquires a collection of skills through these self-generated exploration goals and the associated plans.

The framework is applied to a simulated and actual Katana robot arm manipulating blocks. Results show that our method is able to generate sample-efficient curious exploratory behavior, which exhibits developmental stages, continual learning, and skill acquisition, in an intrinsically motivated playful agent. Specifically, a desirable characteristic of a lifelong learning agent is exhibited: it should gradually move away from learned skills to focus on yet unknown but learnable skills. One particularly notable skill learned, as a by-product of its curiosity-satisfying drive, is the stable placement of a block. Another skill learned is that of stacking several blocks.

## 2. Materials and methods

In this section, we describe the setting of the learning environment, followed by introducing the selective sampling formulation (which is not environment specific). We then describe the planner and the online learning of the world model, and finally present the derivation of the query condition.

### 2.1. Katana in its blocks-world environment

**Figure 1:**
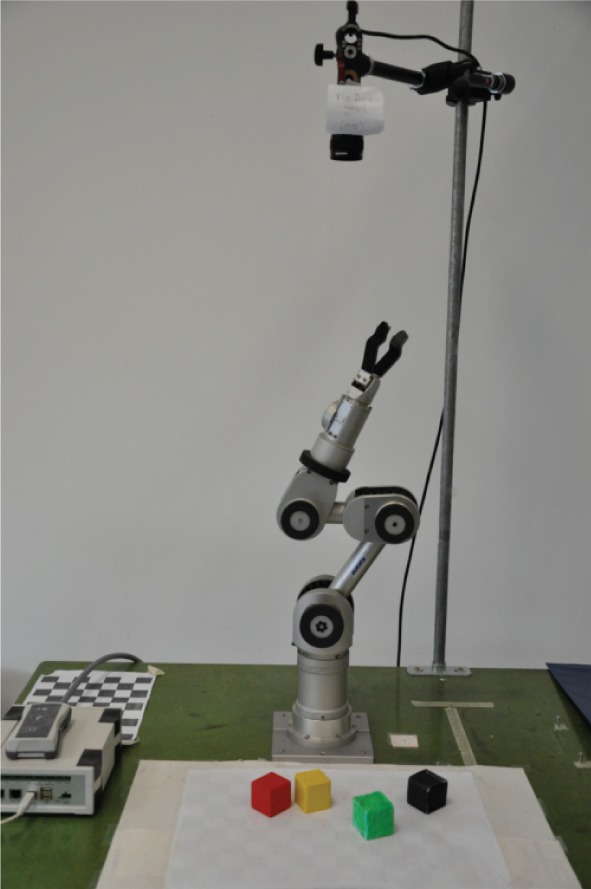
**The Katana robot arm in its blocks-world environment**.

Our robot, a Katana arm (Neuronics, 2004), and its environment, called blocks-world, are shown in Figure 1. There are four different colored blocks scattered in the robot's play area. In Section 3.1 we describe a simulated version of blocks-world with eight blocks. We use the simulated version for a thorough evaluation of our method. In both versions, the agent “plays” with the blocks, through the curiosity-driven exploration framework, and learns how the world works.

In the real-world environment, detection and localization of the blocks is done with straightforward computer vision techniques. The overhead camera was calibrated using the toolbox developed by Bouguet (2009), so that the system can convert 2D image coordinates to the robot's arm-centered Cartesian coordinates. Since all the blocks have different colors, a color-based detection and pixel grouping is used for segmentation, leading to a perceptual system that reliably detects the positions and orientations (in the image coordinate system) of the visible, non-occluded blocks. The positions and orientations of occluded blocks are stored in a memory module. Since any occluded block was once a fully visible block, and the occluded block positions do not change, the memory module updating is also straightforward, requiring basic logic. The purpose of the memory module is to infer the heights of the blocks on top of occluded ones, since the overhead camera does not provide the height information.

When a block is selected for grasping, or a location selected for placement, the system converts the image coordinates to the arm-centered Cartesian coordinates. For reaching and grasping, we use the Katana's inverse kinematics module, which solves for joint angles given the desired pose (position and orientation) of the gripper, and its motion planning module.

In each environment setting, defined as a configuration of blocks, the agent first moves the gripper out of view of the camera, and takes a snapshot of the workspace below. The fundamental choice it needs to make is to decide what the most interesting block *placement location* would be. A placement location is specified by a vector including pixel-coordinates and orientation parameters in the workspace image, as well as the height, in terms of the number of blocks. After the desired placement location is decided, the agent needs to decide which block to pick up for placement. The block that is grasped could be selected via a variety of heuristics. We choose to have the robot grasp the accessible (e.g., non-occluded) block furthest away from the desired placement location, which avoids interference with the blocks at the selected placement location. Grasping will succeed as long as the perception is accurate enough and the block is within the workspace. In the real experiments, grasping is rarely not successful. In these cases, we reset the situation (including internal values related to learning) and have the robot do it again. After grasping, the robot performs another reach, while holding a block, and places it at the desired location.

Next we will illustrate how the robot represents its world, and how this representation leads to something resembling, and which, functionally, serves as an MDP.

### 2.2. Fovea and graph representation

The top-down camera image (640 × 480 pixels) is searched using a subwindow of 40 × 40 pixels, which we call a *fovea*. Each fovea center location represents a possible block placement location.

At any fovea location, the *state s* is the maximum height of a stack of blocks visible in the fovea window. The *action a* is a function of the feature vector that encapsulates the placement location relative to the blocks in any stacks below. How this feature vector is computed will be described below. Any feature vector is converted into one of six possible actions. After an action is executed, i.e., a block is picked and placed at the fovea central location, the *outcome* state *s*' is identified in the same way as *s*, with the fovea location unchanged. The resulting graph resembles a discrete MDP and serves as a basis for tractable exploration in the blocks-world environment.

In a given setting (block configuration), each fovea location maps onto a single (*s*, *a*, *s*') transition in a graph. But only *s* and *a* are visible before the placement experiment. The missing piece of knowledge, which the agent needs to place a block to acquire, is the outcome state *s*'. The fovea can be thought of as a window into a “world” where the robot can do an experiment. Yet, what the robot learns in one “world” applies to all other “worlds.” The question is: which transition is most worth sampling?

Instead of being provided a single state and having to choose an action, as in a classical RL formulation, our system is able to choose one of multiple available state-action pairs from each setting. Availability is determined from the known block positions. The agent's estimated *global state-action value function Q*(*s*, *a*) is used to identify an available state-action pair (s^*^_t_, a^*^_t_) with the highest value, constrained by availability. The agent knows the heights of all blocks in the workspace, which informs it of the possible states currently available. It also knows the fovea location that centers on each block. The desired state s^*^_t_ is selected from the available heights in the current setting, by selecting the one with maximum state value. Next, the desired action a^*^_t_ is selected as the one with maximum Q-value of all action pairings with s^*^_t_. To find a fovea location for the desired (s^*^_t_, a^*^_t_), the agent *searches* by moving the fovea to different placement locations around the stacks of height s^*^_t_, until the contextual information (feature vector **x**_*t*_) associated with the action is matched.

The fovea search occurs in this “top-down” way, since it is computationally burdensome to extract the contextual information of state-action pairs at all fovea positions in each setting. This biased and informed search mechanism is much more efficient. As a future extension, fovea movement would be learned as well [(Whitehead and Ballard, 1990; Schmidhuber and Huber, 1991); see also recent work by Butko and Movellan (2010)].

**Figure 2:**
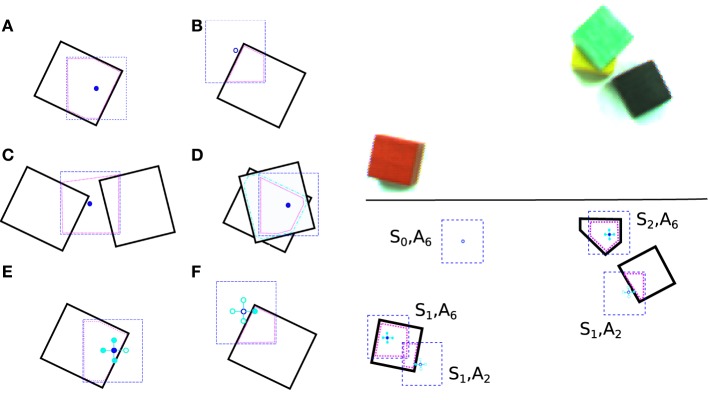
**Left**: (a–f) Examples illustrating the features that were used. **Right**: An example showing how the state and action are encoded (bottom) for a given blocks-world setting (top). See text for details.

Figure 2 (left) shows six examples to illustrate the features used. The thick black lines represent the boundaries of actual blocks. Example fovea locations are represented by the blue dashed squares. The central point of the fovea is shown as a small blue circle. The pink dotted lines show the *convex hulls* constructed from the block pixels *inside the fovea*. If the central placement point is *inside* the convex hull, the feature value is set to one, and zero otherwise. Note the case shown in (c), where the central placement point is not on top of any block at the fovea, but still within the convex hull, and so the feature is set to one. For stacks of several blocks as in (d), the *intersection* of all the block pixels are constructed, and used to construct the convex hull.

As shown in (e) and (f), the features are calculated around the central location, which results in a five-element feature group. In our real robot implementation we use this setup. With a placement location as in (e) four bits are “on,” while in (f), only one bit is “on.” The number of bits that are on, plus one, provides the action index. For example, a fovea location with only one bit on, as described above, would correspond to action *a* = *A*_2_ and is encoded by feature vector **x** = (0, 1, 0, 0, 0, 0). Figure 2 (right) shows an illustration of states and actions at different fovea locations for a particular block configuration. In the lower subfigure, we see the state-action representation underneath a few sampled fovea locations. This representation allows for generalization: the same state-action (*S*_1_, *A*_2_) can be accessed at both the red block (to the lower right) and the black block.

We note in passing that this Katana and blocks-world environment is simplified to become functionally discrete, but the method we use for learning, approximate policy iteration, is not tabular( as the name suggests), nor is the way we use linear basis functions to convert each observation to a feature vector. Our general framework, which will be described next, does not require a tabular environment. Furthermore, the subsystem relevant to a “placement experiment,” i.e., the blocks in the stack right below the fovea, is an MDP according to the formulated graph we use. The approach of considering only relevant features in learning and planning makes the learning, and particularly the planning process, more efficient, as well as tractable^1^.

### 2.3. Selective sampling formulation

Consider an online learning scenario where a learner 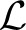 interacts with nature 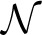 (its environment) in rounds. At each round *i*, nature presents a *setting* 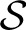
_*i*_. A setting may refer to a single state, or a set of subsystem states (as in our Katana blocks-world environment). Within *each* setting, the learner will observe a sequence of instances **x**_*t*_ ∈ ℝ^*d*^. Here, and for the remainder of this article, we use subscript *i* to denote the setting, and the subscript *t* to denote the instances observed within. Every time the setting is updated, *i* ← *i* + 1, and the observation counter *t* persists (e.g., if there were five instances in setting 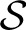
_1_, the first observation in the next setting 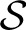
_2_ will be **x**_6_).

For every instance, the learner must decide whether or not to “query” *nature* for the true label *y*_*t*_ of the current instance **x**_*t*_, where *y*_*t*_ ∈ {±1} (for binary classification^2^). By *query* we mean the learner takes an action (*interact with nature*) and observes its outcome. Hence, we can think of **x**_*t*_ as the contextual information associated with each action *a*_*t*_. An observed feature vector, once queried, becomes a training instance to improve the learner. The training will be described in Section 2.5.

Let *Q*_*t*_ ∈ {0, 1} denote the query indicator at time *t*. If a query is issued, i.e., *Q*_*t*_ = 1, the setting is updated (*i* ← *i* + 1), and the learner observes the label of the *queried instance*. It then updates its hypothesis, taking into account the queried example (**x**_*i*_, *y_i_*) as well as the previous hypothesis, which was learned over previous queries. Otherwise, i.e., *Q*_*t*_ = 0, the learner skips the current instance **x**_*t*_ (meaning its label is not revealed) and continues to observe new instances from the current setting (*i* ← *i*).

Clearly, this constitutes a sequential decision process, which generates training examples for the learner. Since each interaction can require the learner to spend time and effort, i.e., labels are expensive to get, it is reasonable to set the objective of the decision process to be such that the learner *learns as much and as fast as it can*.

As a concrete example of this framework, consider our blocks-world environment. Here, a setting is a configuration of all the blocks on the table, while an instance **x**_*t*_ is a feature vector encoding a possible placement location. The fovea sequentially provides possible placement locations, and, for each one, a new instance **x**_*t*_ is observed. For each new instance in turn, the agent predicts the *outcome* of placement. Here, the binary outcome label indicates the success or failure of stacking. The label *y*_*t*_ = 1 indicates a stable placement, while the label *y*_*t*_ = −1 indicates an unstable placement.

After the action is taken, “nature” reveals a new setting 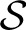
_*i* + 1_ and the agent *obtains*, through observation, the outcome and therefore the label, which will be used to improve its world model. In implementation, the agent obtains the outcome label by comparing two images of the configurations before and after the placement. This is possibly noisy, but usually correct.

### 2.4. Planning in exploration

Our system has a set of adaptive classifiers to predict the block placement outcomes, which, together, constitute the world model 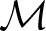. These obtain *knowledge* about the world, and a curiosity-drive causes the agent to desire to accumulate such knowledge (learning progress) as quickly as possible.

**Figure 3:**
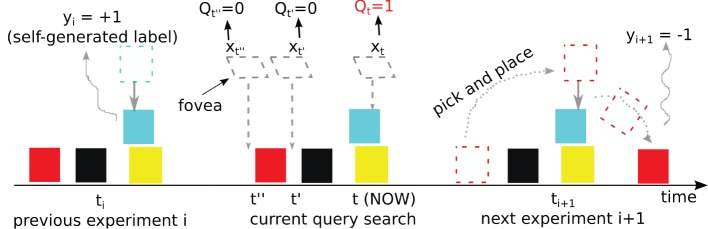
**A single robot-environment interaction, illustrating a setting change**. Each pick and place “experiment” causes a change in setting. The outcome of the previous experiment was that the robot placed the blue block on top of the yellow block, and observed the label +1, corresponding to “stable.” Now (middle), the robot examines three fovea locations (*t*, *t*' and *t*”), each of which involves a query. The query is false for *t*' and *t*”, but true for *t*, and the robot immediately (greedily) grasps the furthest block, which happens to be the red one, and places it at the queried location. The action causes a change in setting to *i* + 1 and the outcome −1 is observed (“unstable”).

The agent is greedy in its pursuit of knowledge. For every instance **x**_*t*_ observed during setting *i*, a *query condition Q*_*t*_ ∈ {0, 1} is generated. The query condition is used to decide if this instance is worth querying for its label (outcome), based on the current model 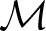
_*t*_ = 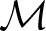
_*i*_. *As soon as it encounters a true query condition, it executes the query*, observes the outcome, and updates the model to 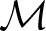
_*i* + 1_. Figure 3 illustrates this exploration behavior in our blocks-world environment.

But in the case where no instances in the setting are deemed to be valuable to query, the agent has to *plan*. In that case, the curiosity drive wants to quickly reach a new setting from which an instance worth querying *can* be observed. To decide which instances are worth querying, the agent simulates future experience of performing different actions from the current setting, and sees, for the simulated new settings, if the query condition becomes true at any point. If so, an intrinsic reward is placed at that transition. A true query condition in simulated experience becomes a binary curiosity reward indicating if an instance is worth exploring. By planning on the *induced* MDP with “phantom” reward function, the agent generates an efficient exploration policy whenever it needs to. These policies for reaching self-generated goals are the skills learned by the agent. Note that this curiosity reward is *instantaneous*, taking into account the current state of the learners, and not a previous learner. See Algorithm 1 for a sketch of this process.

**Algorithm 1:**
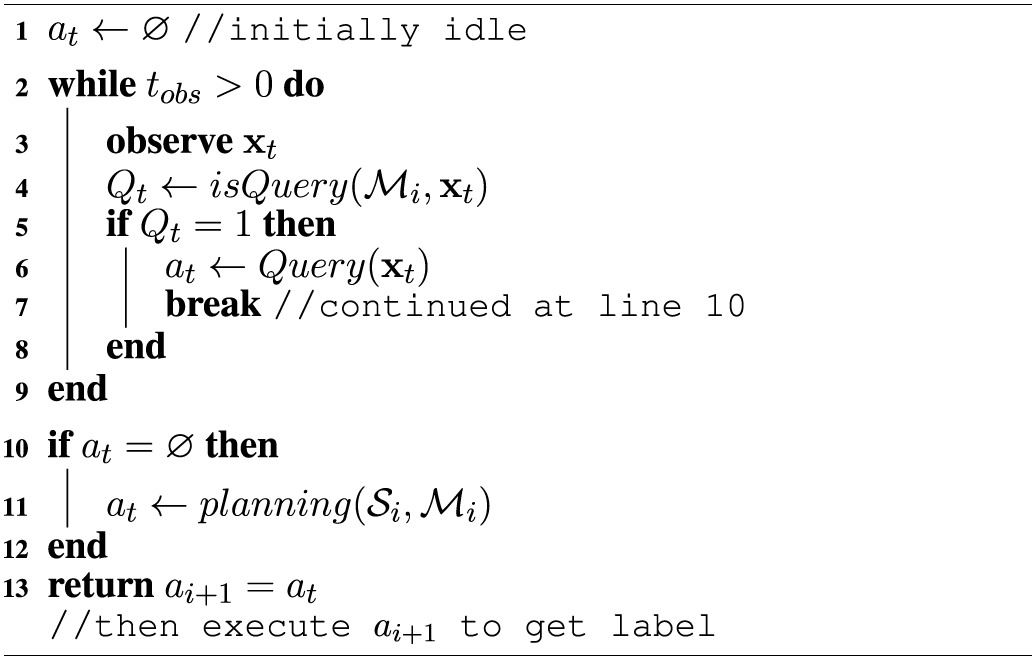
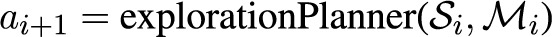

The planner can be implemented using any relevant MDP planning algorithms (Mausam and Kolobov, 2012), for instance, local methods (i.e., for the current state only) like UCT (Kocsis and Szepesvári, 2006), or global methods (for every state) like LSPI (Lagoudakis and Parr, 2003). In our implementation we use approximate policy iteration (LSPI, specifically the algorithm LSTDQ-Model), a global method, to allow the agent to choose between different states/heights (if several stacks are available) in each setting.

In the MDP constructed for our Katana blocks-world environment, the transition probabilities are derived from the adaptive classifiers. At planning time, we update the transition matrix *P*(*s*'|*s, a*) for all state-action-state triplets as follows: *P*(*s*'|*s, a*) = 0 if *s*' > *s* + 1; *P*(*s*'|*s, a*) = (1 + $\hat{\Delta }$)/2 if *s*' = *s* + 1; and *P*(*s*'|*s, a*) = (1 − $\hat{\Delta }$)/2/*s* if *s*' ≤ *s*, with the prediction margin $\hat{\Delta }$ computed as the inner product between the contextual feature **x** representing action *a*, and the linear weight vector **w** of the predictor, i.e., $\hat{\Delta }$ = **w** · **x** (more details will be provided in the next section). In other words, the transition probability to current height plus one is equal to the probability of a stable placement. It is zero for any height which is two or higher above the current one, and is a uniform fraction of the probability of instability for the lower heights. Note that this is just an approximation, but it is good enough for effective planning to reach higher heights.

The next two sections describe our adaptive learners and the derivation of query condition, based on these learning models.

### 2.5. Online learners

We focus on adaptive binary linear classifiers. There are multiple such classifiers in our system—one per height—but the discourse in this subsection will be with respect to a single classifier, for simplicity. For such a classifier, with weight vector **w**_*t*_ ∈ ℝ^*d*^, a classification of instance **x**_*t*_ is then of the form *ŷ_t_* = sign (**w**_*t*_ · **x**_*t*_). The term $\hat{\Delta }$_*t*_ = **w**_*t*_ · **x**_*t*_ is often referred to as the *prediction margin* attained on instance **x**_*t*_, and the magnitude of the margin |$\hat{\Delta }$_*t*_| is a measure of confidence of the classifier in label prediction^3^.

In the setting of a developmental robot interacting with nature, *training* instances are generated in a biased manner. They are not independent and identically distributed—the sampling/query process depends on the learner's adaptive model 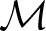
_*t*_. However, their corresponding labels can be assumed to be generated from a linear stochastic model. Specifically, we make the following assumptions: 1) The labels *y*_*t*_ ∈ {−1, +1} are realizations of independent random variables *Y*_*t*_ sampled from a stochastic source with a probability density function *P*(*Y*_*t*_ |**x**_*t*_) continuous at all **x**_*t*_. This entails that, if Δ_*t*_ = 𝔼[*Y*_*t*_ |**x**_*t*_] ∈ [−1, 1], then sign(Δ_*t*_) is the Bayes optimal classification. 2) There exists a fixed but unknown vector **u** ∈ ℝ^*d*^ for which **u** · **x**_*t*_ = Δ_*t*_ for all *t*. Hence **u** is the Bayes optimal classifier under this noise model.

Note that when running our algorithms in a reproducing kernel Hilbert space (RKHS) 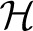 with a universal kernel (Steinwart, 2002), the classifiers are implicitly non-linear, and Δ_*t*_ is well approximated by *f*(**x**_*t*_), for some non-linear function *f* ∈ 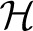, hence assumption 2 becomes quite general.

The key elements in designing an online learning algorithm include the comparator class 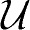 ⊆ ℝ^*d*^, the loss function ℓ, and the update rule. For an arbitrary classifier **v** ∈ 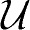, denote by ℓ(**v**; **x**_*t*_, *y*_*t*_) its non-negative *instantaneous* loss suffered on the current example (**x**_*t*_, *y*_*t*_), and abbreviated by ℓ_*t*_(**v**), i.e., ℓ_*t*_(**v**) = ℓ(**v**; **x**_*t*_, *y*_*t*_). We define the total loss of an *adaptive* learner 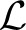 on a particular sequence of examples 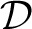 = {(**x**_*t*_, *y*_*t*_)}^*T*^_*t* = 1_ as 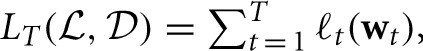, and we also define the total loss of some (fixed) classifier **v** as 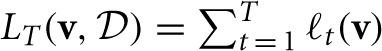. A good learner that makes few online prediction mistakes also has small *relative* loss compared to the best linear hypothesis **u**:

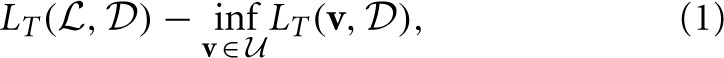



for any sequence 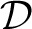. Since the online learner only observes one example at a time, the relative loss is the price of hiding future examples from the learner (Azoury and Warmuth, 2001). A desired analysis step in designing online learners is then to prove upper bounds on such a relative loss. This bound should grow sublinearly in T, so that it vanishes when T approaches infinity.

We use a modified version of the widely used regularized least square (RLS) classifier (Azoury and Warmuth, 2001; Cesa-Bianchi et al., 2009; Dekel et al., 2010)—a variant of the online ridge-regression algorithm—as our online learner. As the name suggests, this class of algorithms uses the squared loss function, and possesses a proven relative loss bound under our label noise model (Vovk, 2001; Dekel et al., 2012), with the desired sublinear growth. Established results for the algorithm will be used to derive our query condition (Section 2.6).

Given the sequence of queried (i.e., training) examples up to setting *i*, {(**x**_*j*_, *y*_*j*_)}^*i*^_*j* = 1_, the RLS classifier maintains a data correlation matrix, $A_{i}=I+\sum_{j=1}^{i-1}x_{j}x_{j}^{⊤}$, with *I* the *d* × *d* identity matrix and *A*_1_ = *I*. For the *i*-th queried instance **x**_*i*_, the weight vector can be updated as **w**_*i* + 1_ = *A*^−1^_*i* + 1_(A_*i*_**w**_*i*_ + *y*_*i*_**x**_*i*_).

The inverse matrix *A*^−1^_*i* + 1_ can be updated incrementally using the Sherman-Morrison method,

$$A_{i+1}^{-1}=A_{i}^{-1}-\frac{b_{i}b_{i}^{⊤}}{1+c_{i}},$$

where

$$b_{i}=A_{i}^{-1}x_{i}$$

and

$$c_{i}=x_{i}^{⊤}A_{i}^{-1}x_{i}=x_{i}\cdot b_{i}.$$

Using the fact that *A*^−1^_*i* + 1_ **x**_*i*_ = **b**_*i*_/(1 + *c*_*i*_), the weight vector update is simplified as:

$$w_{i+1}=w_{i}+\frac{\left(y_{i}-w_{i}\cdot x_{i}\right)}{1+c_{i}}b_{i}.$$

An implementation-efficient pseudocode of this modified RLS update rule is presented in Algorithm 2.

**Algorithm 2:**
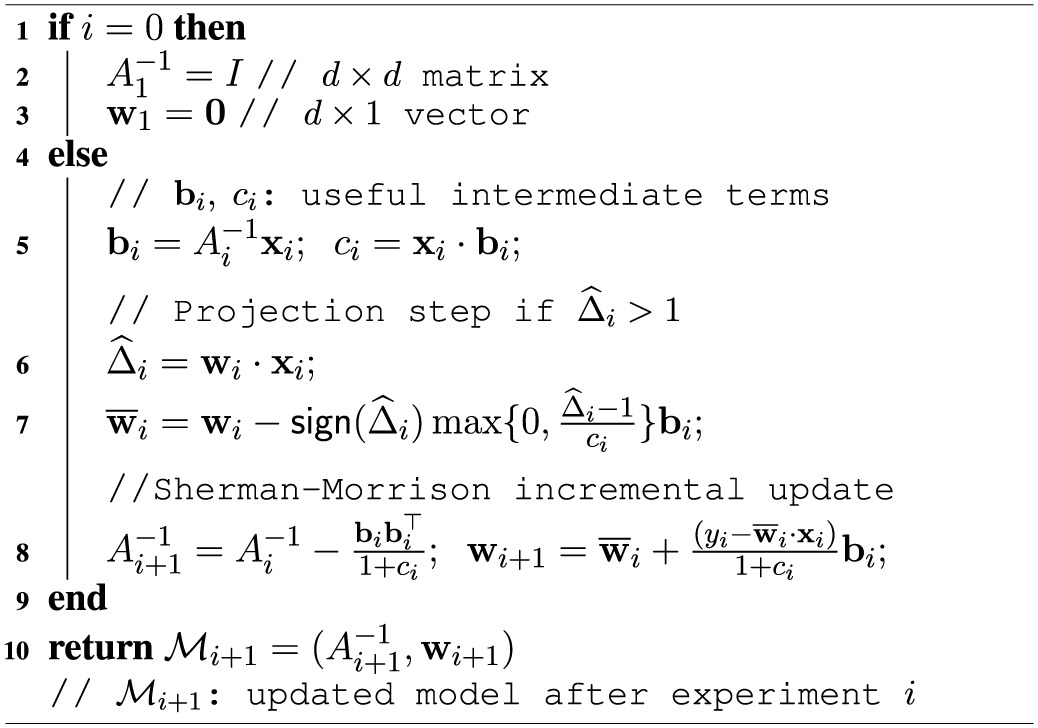
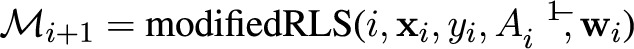

### 2.6. Query condition

Our query condition is greatly inspired by work in selective sampling, a “stream-based” setting of active learning (Atlas et al., 1989; Freund et al., 1997). In selective sampling, the learner has access to an incremental stream of inputs and has to choose, for each datum in order, whether to query its label or not. State of the art methods in selective sampling, with theoretical performance guarantees, include BBQ (Orabona and Cesa-Bianchi, 2011) and DGS (Dekel et al., 2012). These methods also use variants of the RLS algorithm (Azoury and Warmuth, 2001; Vovk, 2001; Auer, 2003; Cesa-Bianchi et al., 2005; Cesa-Bianchi and Lugosi, 2006; Cavallanti et al., 2008; Strehl and Littman, 2008; Cesa-Bianchi et al., 2009), and maintain a data correlation matrix to calculate a confidence interval or uncertainty level in their prediction, which is essentially an estimate of the variance of the RLS margin for the current instance.

The query condition must indicate when the outcome is statistically known or unknown. Here we derive a query condition for this purpose, based on the expected learning progress. Essentially, when the learner is certain in what it predicts, it can ignore the instance, since, with high probability, its learning model will not get updated much on this example if it is queried. Inversely, only those instances that the learner is uncertain in its prediction are worth querying for labels, since the model of the learner will undergo a large update on such training examples.

The following lemma from Orabona and Cesa-Bianchi (2011) defines χ_*t*_, the *uncertainty level*, or *confidence interval* of the RLS prediction.

**Lemma 1**. Let δ ∈ (0, 1] *be a confidence level parameter*, *h*_δ, **u**_(*t*) *be a function of the form*

$$h_{\delta ,u}(t)=||u|{|}^{2}+4\sum_{k=1}^{i}r_{k}+36\log\frac{t}{\delta },$$

*where* ||**u**|| *is the unknown squared norm of the optimal Bayes classifier, and r*_*i*_ = **x**^⊤^_*i*_ *A*^−1^_*i* + 1_ **x**_*i*_.

*Now, define* $\chi_{t}=\sqrt{c_{t}h_{\delta ,u}(t)}$ with *c*_*t*_ = **x**^⊤^_*t*_ *A*^−1^_*i* + 1_ **x**_*t*_. *With probability at least 1 − δ, the following inequality holds simultaneously for all t*:

$$|\Delta_{t}-{\hat{\Delta }}_{t}|\leq \chi_{t}.$$

This inequality can be rewritten as,

$$\Delta_{t}{\hat{\Delta }}_{t}\geq \frac{\Delta_{t}^{2}+{\hat{\Delta }}_{t}^{2}-\chi_{t}^{2}}{2}\geq \frac{{\hat{\Delta }}_{t}^{2}-\chi_{t}^{2}}{2},$$

which essentially implies that if |$\hat{\Delta }$_*t*_| > χ_*t*_, the learner is **certain** (with probability at least 1 − δ) that $\hat{\Delta }$_*t*_ and Δ_*t*_ have the same sign (i.e., $\hat{\Delta }$_*t*_ Δ_*t*_ > 0), and **there is no need to query for the true label**. Inversely, when |$\hat{\Delta }$_*t*_| ≤ χ_*t*_, the learner is **uncertain** about its prediction, and **it needs to issue a query**. Formally, the query condition is stated as follows:

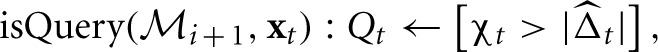



where [·] denotes the indicator function of the enclosed event.

Now, from Lemma 1 we also have |Δ_*t*_| ≤ |$\hat{\Delta }$_*t*_| + χ_*t*_. Combined with the query condition derived above, we have | Δ_*t*_| ≤ 2 χ_*t*_ with probability at least 1 − δ when a query is issued. When the magnitude |Δ_*t*_| of the optimal prediction margin is small, the instance label is almost certainly noise, i.e., the prediction is nearly a random guess. These instances are “hard” or even “impossible” to learn, and the learner should instead focus on other instances that it can improve its prediction capability. We derive another query condition to reflect this insight, by enforcing another threshold θ on the uncertainty level,

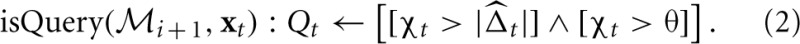



In implementation, a surrogate or proxy function is used to avoid dependency on the optimal yet unknown **u**. This takes the form,

$$\chi_{t}=\alpha \sqrt{c_{t}h(t)},$$

where α is a tunable positive parameter, and

h(t)=log(1+i)

is a simplification of *h*_δ, **u**_(*t*). Importantly, the confidence interval does not depend on the squared norm of the optimal but unknown Bayes classifier **u**. See Dekel et al. (2012) Equation (12) and Lemma 7, notice the additional assumption of ||**u**|| ≤ 1. See also Orabona and Cesa-Bianchi (2011) Algorithm 2 for another proxy function.

## 3. Results

In all implementations we used the following parameter values: discount factor γ = 0.95, and query condition scaling factor α = 1. The confidence-interval threshold θ = 0.01 for simulations, while θ = 0.1 was used in the real robot experiments.

### 3.1. Simulated blocks-world environment

We designed a stripped-down simulated version of the actual blocks-world, in order to test our system. In simulation, thousands of trials can be run, which would take far too long on the real robot. Of course we cannot capture all aspects of the real-world robot setting, but we can capture enough so that the insights and conclusions arising from simulated results suffice to evaluate our system's performance.

The simulated environment also allows us to use any number of blocks and any number of features. For any configuration of blocks, some set of heights will be available for the agent to place upon, corresponding to the heights of the top blocks in the stack(s), and height zero. In the simulation, we use eight blocks, and 21 features. Each height's feature vector is of length 21 bits, with only one bit set. All 21 feature vectors are available for each available height. The agent must select one of them. Unlike the actual robot setting, in simulation, the features do not correspond to any physical aspect of the simulated world. In simulation, each of the 21 features are associated with a different probability of stability, which is randomly generated.

Each possible height *s* has a *different* weight vector **u**^*s*^, which is the randomly generated “true model” for the result of placing a block upon it. This was done in order to generate simulated block placement outcomes in an easy-to-implement way. There are 21 components^4^ of each **u**^*s*^, which are randomly generated in the range [ −1, 1]. An outcome (stable/falling) is generated using the corresponding height's true (probabilistic) model, where the actual outcome label sign(**u** · **x**_*t*_) is flipped with probability $\frac{1-|u\cdot x_{t}|}{2}$. For the purpose of generating orderly plots in Section 3.2, we re-order the 21 feature vectors of each height in ascending order of their likelihood of stability, then re-assign their feature indices from 1 to 21. Thus, the smaller the feature index, the lower likelihood the placement will be stable. For an outcome of falling, there is a chance that the entire stack underneath the placement position collapses, in which case all blocks in that stack are reset to height one.

The eight blocks' configuration is represented by vector **q**. The absolute value of each element |*q*_*j*_| is the height of the corresponding block *j*. We set sign(*q*_*j*_) = −1 if block *j* is occluded (stacked upon), while sign(*q*_*j*_) = 1 means block *j* is on the top of its stack, which means its both graspable and another block can be placed upon it. The set of different positive elements of **q** constitute the set of current available states (heights to place upon) in addition to height zero (which is always available). For example, vector **q** = (−1, −2, −3, 4, −1, 4, −2, −3) means the configuration has two different stacks of height four, having block IDs 4 and 6 on top of the two stacks. Here, the set of available placement heights is height zero and height four.

After selecting the state and action, the agent picks an “available” top block, and “places it.” By available, we mean it is the top block of another stack. Another block in the stack (if any) of the block that is grasped becomes a top block. If the placement is stable, the highest block in the placement stack has its sign reversed, and the placed block becomes the top block of that stack. If the placement outcome turns out to be unstable, a “toppling” event occurs, where one randomly selected block in the stack of placement, with a lower height, becomes a top block of the remaining stack, with blocks below unchanged. The (unsuccessfully) placed block and the other, higher blocks in the stack topple to the surface, and their values are all set to +1.

### 3.2. Results in simulated blocks-world

**Figure 4:**
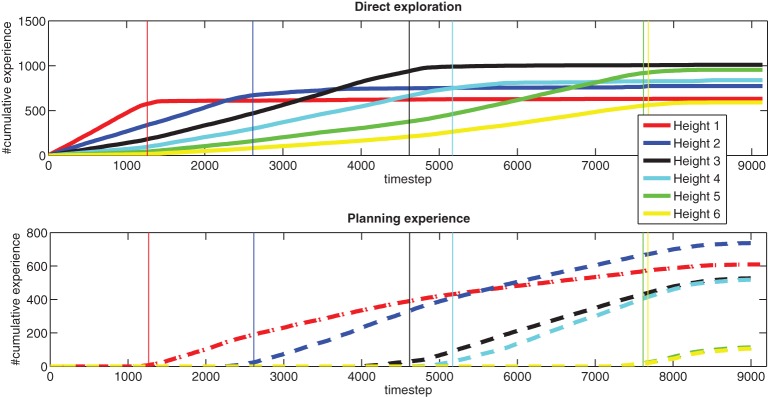
**Exploration history (averaged over 10 runs)**.

Figure 4 shows the *averaged* exploration behavior of our system over time, for all different heights. “Direct exploration” refers to settings where the query condition is true, while “planning experience” refers to settings where the algorithm has to execute a planned action (since the query condition is always false for that setting). On the *y*-axis, “cumulative experience” is a count of the number of times these types of actions are generated. The different colored lines indicate different heights. The vertical lines are from a single run, and indicate when, during that run, the learner switches from direct exploration of one height to planning exploration of higher heights.

These plots show the developmental stages of the learning agent, where easier problems, such as direct exploration at height one, are learned first, and more difficult problems are learned later. They also show cumulative learning, as the acquired knowledge at lower heights is exploited for planning, and this planning helps the agent get to the higher heights, in order to acquire more knowledge. The difficulty of this problem is shown by the time the learner needs to spend to fully explore its environment, especially in achieving the highest heights. For instance, to even get to height six to do experiments, the agent first needs to stack blocks from lower heights each time the stack collapses, which is a regular occurrence.

The agent does not necessarily explore a single height until everything at that height is statistically known. There are sometimes situations where several heights worth exploring are available simultaneously in the environment. In such cases, the agent starts with the height having the largest “future exploration value” as estimated by LSPI. The planning step helps to trade off “easy-to-get” small learning progress rewards with “harder-to-get” larger ones. As shown in Figure 4, the exploration at higher heights does, in fact, start before the direct exploration of lower heights terminates.

**Figure 5:**
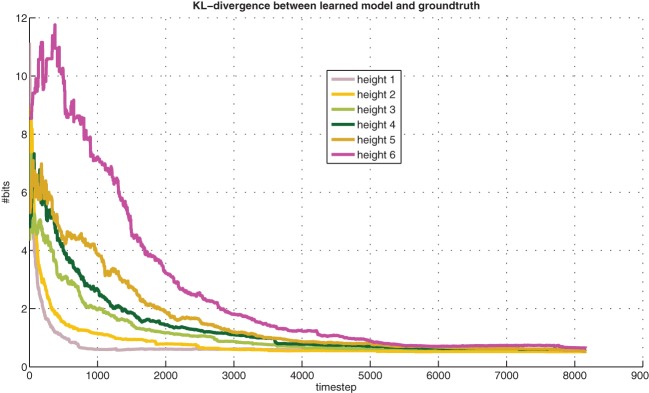
**KL-divergence between learned models and ground-truth models (averaged over 10 runs)**. Best viewed in color.

Figure 5 shows the learning progress, measured with Kullback-Leibler (KL) divergence between the learned models and the true models. These distances tend to diminish exponentially with experience, and they diminish faster at lower heights, where experience is easier to get. When each line in the graph saturates, it corresponds to the associated knowledge being “known” and ready for exploitation in planning. The saturation levels are non-zero due to the noise level in the training labels, the query condition scaling factor α, and the confidence-interval threshold θ.

**Figure 6:**
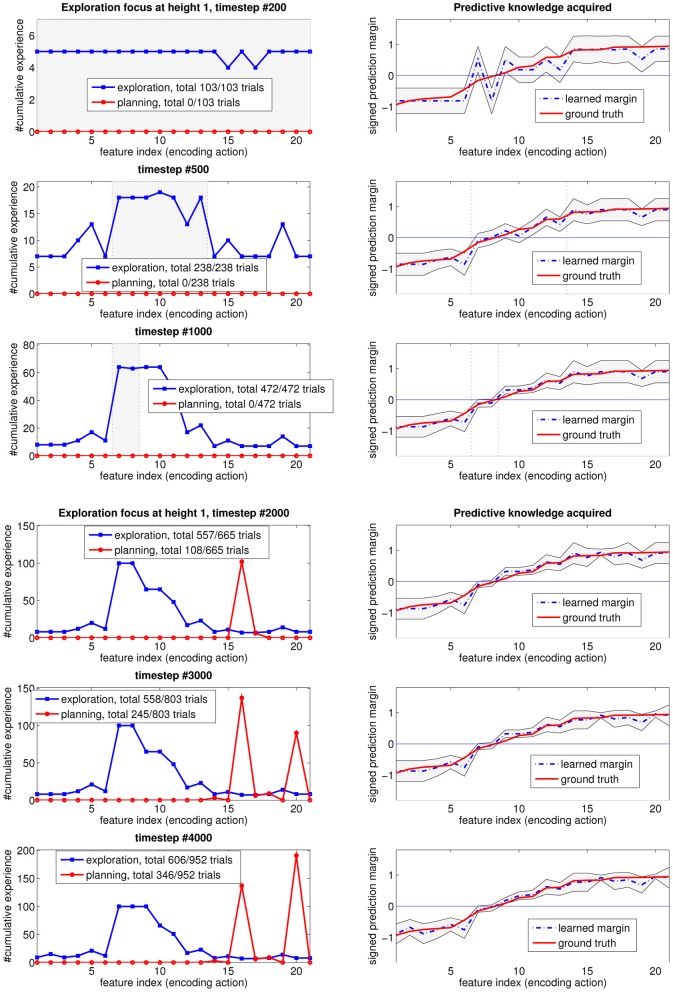
**How the focus of the self-generated exploration goals at height 1 changes over time as the learned predictive model gets closer to the true one**.

Figure 6 shows how the *exploration focus* changes over time, for height one. In each subgraph row, the figure on the left shows the distribution of the experience up until the timestep in the subfigure title. The shaded area between the two vertical lines represents the “unknown” region of input features that is deemed to still be worth exploring. This will be the “exploration focus” of the agent, in subsequent interactions. Regions outside of this shaded area are considered “known” by the learning agent, and not worth exploring any more. Going from the top to the bottom of Figure 6, note that the query region shrinks with the amount of experience. Additionally, note that the middle features, associated with the most uncertain outcomes (as mentioned in Section 3.1) stay interesting longer than the others.

An interesting observation that is worth elaborating on, is as follows. At timestep #2000, when every prediction is statistically known, the agent starts to exploit the acquired knowledge for planning (i.e., taking its estimated “best” action #16 to reach height two). It also keeps on refining the learned model, which reveals, as a result of generalization in learning that the optimal action (i.e., the most stable placement position) is action #20 instead. Afterwards, the agent switches its optimal policy for this height, as shown in timesteps #3000 and #4000.

The plots on the right shows the learned predictive model (blue dashed lines), with two thin black lines representing the confidence intervals for each prediction. As more data are observed, the associated confidence interval will shrink, reflecting the learning progress. Note that as a result of generalization, the neighboring area of the input feature space also gets improved, indirectly, in its confidence interval. Recall that we re-arranged the input feature indices so that their prediction margin (hence, probability of stable/unstable outcomes) are in ascending order.

A measure of the difficulty of a learning problem is the sample complexity needed to achieve some desired level of confidence. The shaded regions (i.e., “unknown” and worth exploring) shrink with experience, toward the input feature values with small prediction margin ground-truth. These feature values correspond to the input subspace with prediction outcomes close to noise, i.e., hard to predict. However, these instances lying close to the decision boundary are the most informative instances for constructing a good decision plan. Our system first explores much of the input space, then quickly shifts its attention to this “hard-to-learn” input region, where most of its exploration effort is spent. As a result, the learned predictive model gets closer to the true model over time. Note that for “known” regions outside the shaded area, even though the number of experiences is small, and the confidence interval (i.e., uncertainty level) is large, the learning algorithm is still confident that its prediction (sign of the margin) is close to the optimal one with high probability. Thus, these regions are not worth exploring any more.

**Figure 7:**
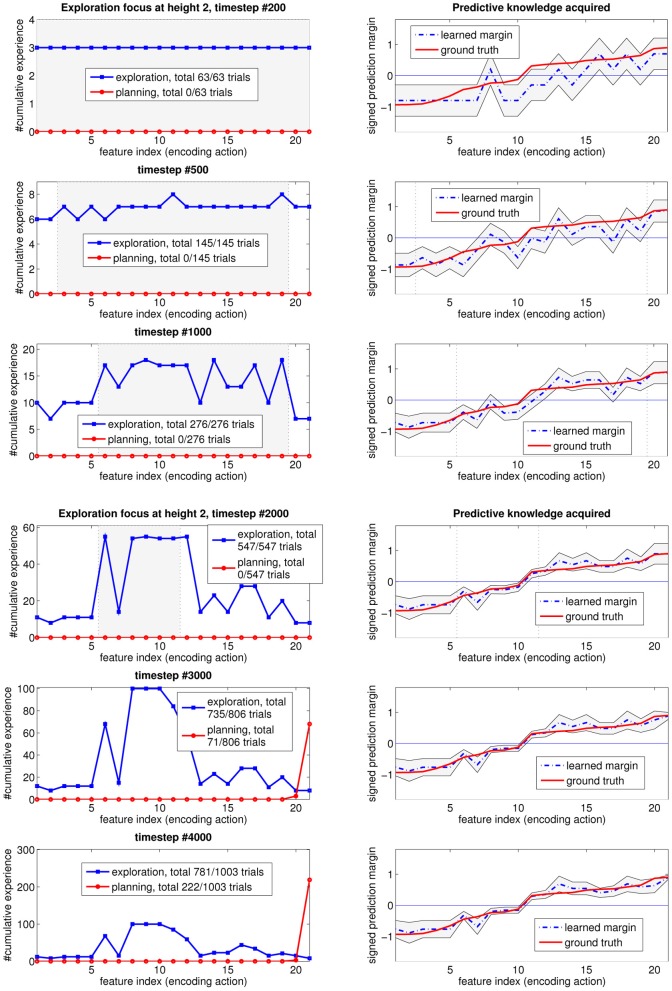
**How the focus of the self-generated exploration goals at height 2 changes over time as the learned predictive model gets closer to the true one**.

**Figure 8:**
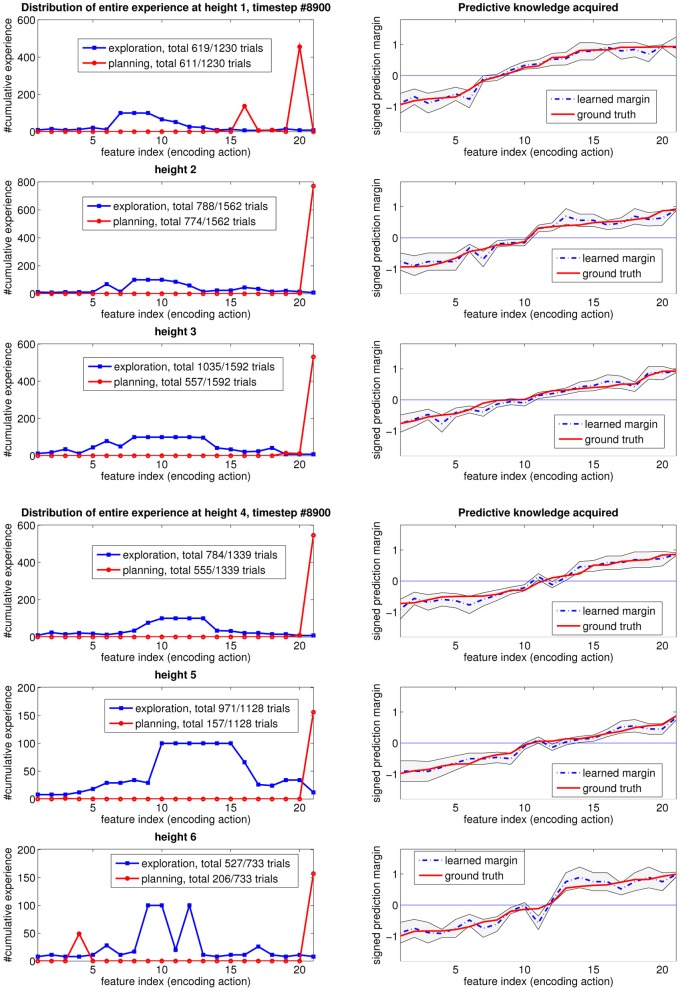
**Experience distribution after the last timestep (learning has completed) for heights 1–6**.

The same exploration behavior is observed when we analyze the data for other heights, as shown in Figure 7 for height two, and Figure 8 for the first six heights when exploration terminates. In all the experiments, the agent first explores the whole input feature space, then focuses on subspaces of input features that are informative but for which high confidence is hard to achieve, then on features that are useful for planning. This typically occurs for each height in turn. As a result of learning how to plan, which necessarily entails reliably transitioning from one state (height) to another, the skill of block stacking is achieved.

To further analyze the effectiveness of our method, we compare its performance to three other methods. The comparison measure is the KL-divergence with respect to the true model. The first method simply is uniform random action selection, which results in undirected, babbling-like, behavior. The second method, which we call Conf (Ngo et al., 2012), uses confidence intervals χ_*t*_ of the prediction margin directly as phantom rewards to generate the exploration policy through planning. Intuitively, this is also an informed exploration method since it promotes exploration in parts of the environment with high uncertainty. The main difference is the confidence intervals are used themselves as rewards, instead of using a query condition. The third method is a variant of our proposed method, but the exploration policy is updated (i.e., planning) after every 10 observations, instead of on-demand whenever exploration planning is invoked. We denote this variant as Q10, and our proposed method as Q1.

**Figure 9:**
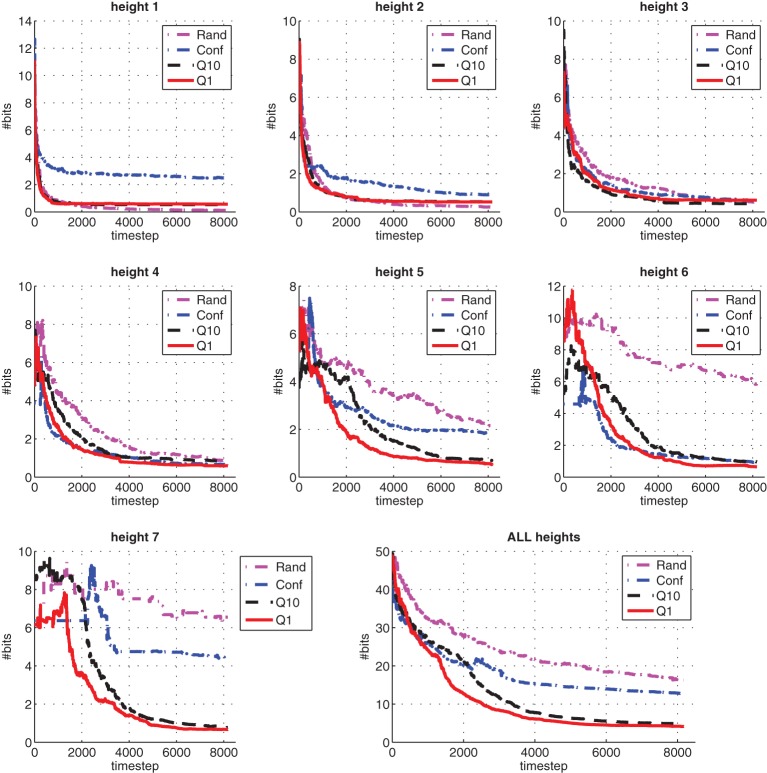
**A comparison of exploration methods in terms of the KL-divergence between the learned predictive models at each time step and their ground-truth models**. Results are averaged over 10 runs.

The results are shown in Figure 9, with each subgraph showing the KL-divergence between learned models and their ground-truth at each timestep. Inspecting carefully the subgraph for height one and two, we see that Q1 gets close to the true model exponentially fast in the first 1000 timesteps, then saturates. The random method, on the other hand, though making much slower progress than Q1 and Q10 in the first 1000 timesteps, keeps improving its learned models and achieves the best models for height one and two, among the four methods. However, for the other five higher heights, its learned models are much worse compared to the rest. This can be explained by the fact that the blocks-world environment naturally generates unbalanced experience distribution among all the states under random action selection, and lower heights will get much more learning experience compared to higher ones. This undirected exploration behavior makes random exploration the least efficient method compared to the other three (informed) exploration methods, as shown in the overall results in the last subgraph at the bottom-right corner. The confidence-based method performs much better than random method, but is still inferior compared to query-based methods Q1 and Q10. The overall performance of Q1 is the best, closely followed by Q10, which is less efficient due to less frequent planning updates.

### 3.3. Results on the real robot

**Figure 10:**
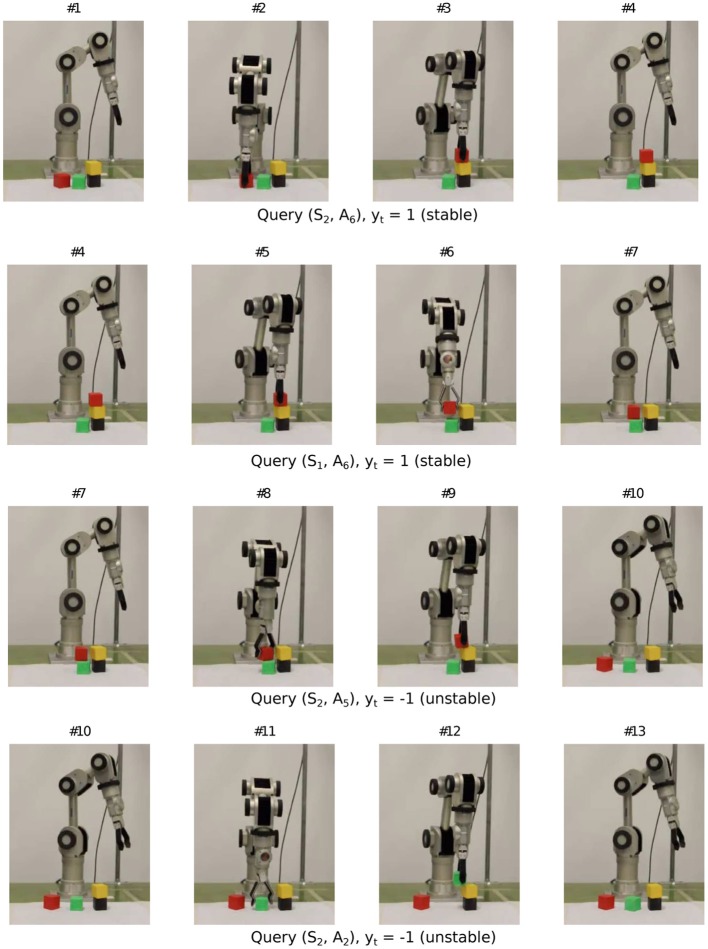
**Sample query sequence on real robot (1/3)**.

**Figure 11:**
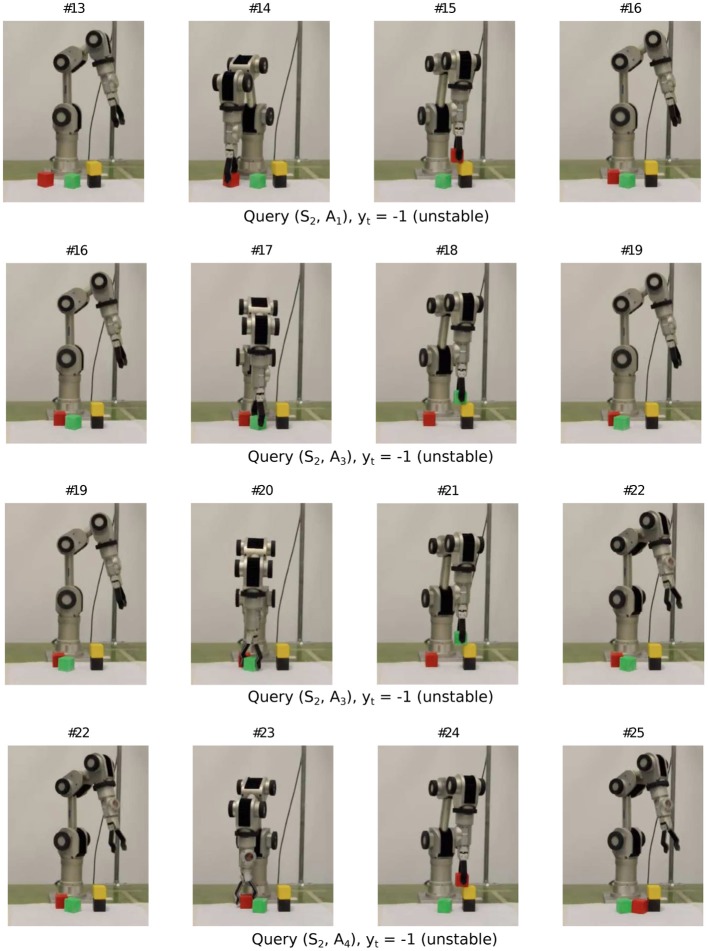
**Sample query sequence on real robot (2/3)**.

**Figure 12:**
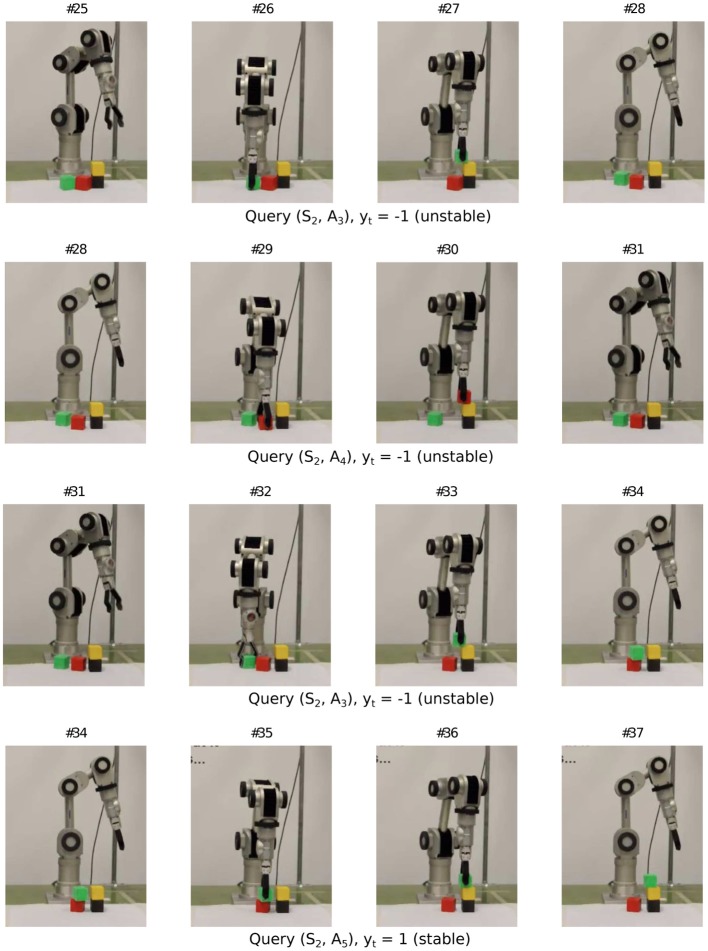
**Sample query sequence on real robot (3/3)**.

Now, we show the learning behavior on the real robot. Figures 10–12 show a snippet of experience consisting of 12 consecutive experiment sequences. In each frame, one should focus on the configuration of the blocks in the workspace and track the changes from the previous frame. Each sequence starts with i) a fovea-based search for the desired placement in the current block configuration (i.e., either the query condition returns “unknown” or the best planned action is selected), as shown in the first column, followed by ii) an action picking a block unrelated to the placement experiment (second column), then iii) placing the block at the desired height, orientation, and relative position with respect to the stack below (third column). The sequence ends with an observation process to self-generate the label (last column). The end of one sequence is also the beginning of the next sequence. Since the robot has already had some prior experience before continuing from sequence #1 of the snippet, it now focuses on exploring height two. Specifically, from all the 12 sequences, we find that the robot gradually shifted its attention (from the second sequence in Figure 11 to the second last sequence in Figure 12 to trying actions *A*_3_ and *A*_4_ (corresponding to relative placement positions with two and three bits set), which are actually the actions with the most uncertain placement outcomes among the six actions. Note that with tower height four, the robot arm does not have many feasible workspace points for the pick and place task. Hence we limit the maximum height to three.

**Figure 13:**
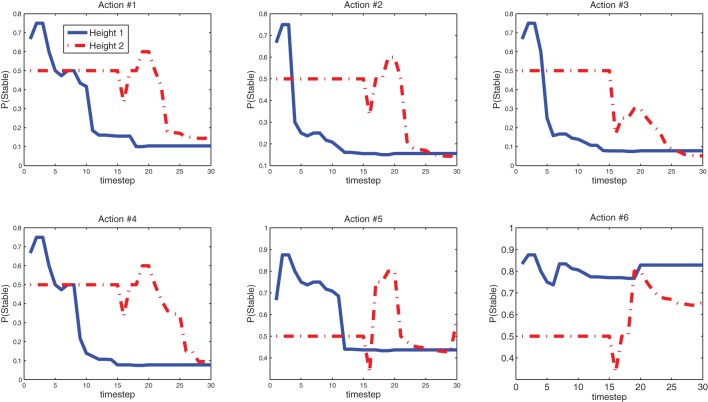
**Learning progress of the Katana robot arm's predictive models at height 1 and 2 after 30 settings**. Action 1 (no bits set) is the most unstable. Action 6 (all bits set) is the most stable. See earlier discussion on the features and Figure 2.

Figure 13 shows the predictive models the Katana robot arm acquired in a single run with 30 interactions (see demo video at www.idsia.ch/~ngo/frontiers2013/katana_curious.html; the last 12 interactions shown in Figures 10–12 start from 1:52).

**Figure 14:**
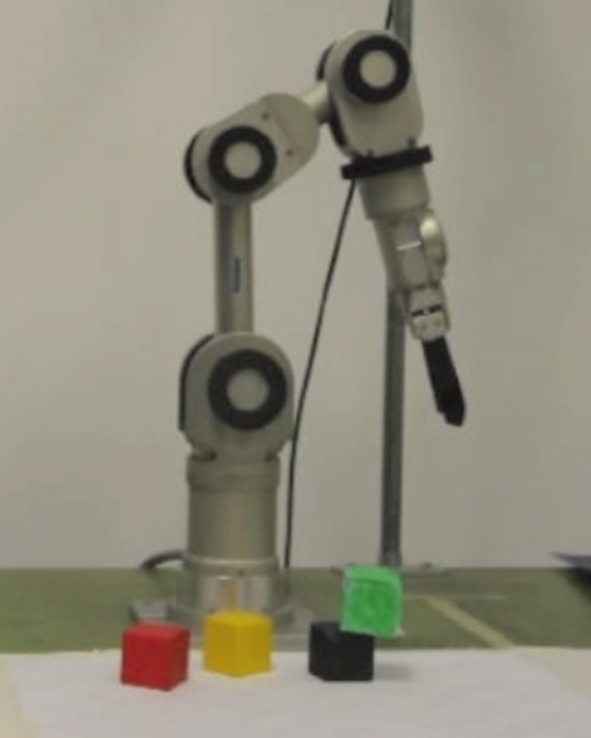
**A “tricky” situation to test the robot's stacking skill**. We show this case to illustrate the value of exploring to learn how the world works. Consider the robot is faced with a task to build a stack of blocks as fast as possible from this initial setting. Given its learned model of the world, the robot will decide to start stacking from height 1 instead of height 2, as with high probability the stack of two blocks will fall after placing another block upon them.

Figure 14 shows a “tricky” situation for the robot, which it can overcome if it has learned the model well. Here, the robot must demonstrate its block stacking skill, as an externally imposed goal.

## 4. Discussion

### 4.1. Systematic exploration

This work was conceived with *pure* exploration in mind, which is contrasted with the treatment of exploration in classical RL. There, exploration is discussed in terms of the *exploration-exploitation tradeoff*. On the one hand, the agent should *exploit* the acquired knowledge by selecting the current best (greedy) action, thereby not spending too much time in low-value areas of the state space. On the other hand, it needs to *explore* promising actions to improve its estimation of the value function, or to build a more accurate model of the environment.

The most widely used method for balancing exploration and exploitation is the ϵ-greedy algorithm (Watkins and Dayan, 1992; Sutton and Barto, 1998). At each state, with probability of 1−ϵ the agent selects the greedy action with respect to the estimated value function, and with a small probability of ϵ it selects a *random* action for exploration. *Optimistic initialization* is another common method for exploration (Sutton and Barto, 1998). By initializing the value function for all states with high values, the agent will try to reach less visited states until their values converge to near-optimal ones, which is much lower than the initial values. The initial values strongly affect the exploration time. Progress-driven artificial curiosity is a more general method for balancing exploration and exploitation which 1. removes the reliance on randomness—the exploration is *informed*, instead of relying on randomness (uninformed), and 2. promotes exploration of states where learning can occur over states where not much can be learned. To contrast, in optimistic initialization, every state is equally worth exploring.

Somewhat recently, several algorithms modifying optimistic initialization have been proposed that guarantee to find near-optimal external policies in a polynomial number of time steps (PAC-MDP). These algorithms, such as *E*^3^ (Kearns and Singh, 2002) and R-max (Brafman and Tennenholtz, 2003), maintain a counter for the number of times each state-action pair is tried. When this number exceeds some threshold, the estimated state-action value is quite accurate, and the state-action pair will be considered “known”—thus with high probability the greedy action will be near-optimal (exploitation). Otherwise, the value is replaced with a highly optimistic one, encouraging the agent to explore such “less-selected” state-action pairs. Recent work in this model-based line of research extends R-max in several aspects. Rao and Whiteson (2012) give a better estimate of the optimistic reward using a weighted average between experienced and optimistic ones, resulting in the V-MAX algorithm that is capable of exploiting its experience more quickly. Lopes et al. (2012) propose to replace the counter of visits to a state with expected learning progress based on leave-one-out cross-validation on the whole interaction history. Our method for estimating learning progress is, in contrast, instantaneous and online. Furthermore, it is able to generalize across different actions, instead of treating them separately.

The common theme in many intrinsically motivated RL approaches is that the estimated learning progress is used as secondary to external rewards. The purpose of the behavior (i.e., the policy) of the agent has a goal of achieving external rewards. Exceptions include, for instance, Şimşek and Barto (2006), where the agent's behavior is based on a second value function using an intrinsic reward signal, which is calculated based on the changes in value estimates of external rewards.

Besides our preceding work (Ngo et al., 2012), which this work is an extension of, some recent work in the pure exploration setting also uses planning. Yi et al. (2011) develop a theoretically optimal framework based on the Bayesian methods, in which the agent aims to maximize the information gain in estimating the distribution of model parameters. An approximate, tractable solution based on Dynamic Programming is also described. Hester and Stone (2012) present results on simulated environments, where *two* progress-based intrinsic reward signals are used for exploration: one based on the variance in predictions of a decision tree model, and one based on the “novelty” of the state-action pair, to promote the exploration focus to shift toward more complex situations. In our system, we use a *single* curiosity reward signal based on the derived query condition, and our approach has been shown to be more effective than the previous variance-based approaches, since observations with large variance will not be worth querying *if* the learner is confident about its predictions.

In all the aforementioned work with pure exploration, planning is used to generate exploration policies, which must be invoked at every timestep. It has been observed (Gordon and Ahissar, 2011; Luciw et al., 2011) that quickly learning agents do not update their exploration policies fast enough to achieve the intrinsic rewards they expect to achieve. In such cases, learning progress-based exploration is no better than random action selection or various simple heuristics. In other words, the update speed of the policy generation must be much greater than the learning speed of the underlying learner. This can be computationally demanding. It can also be wasteful, when the intrinsic reward that the agent plans to achieve is, while non-zero, quite small.

Our approach allows the agent to choose the most informative observations (possibly several steps ahead) to sample, and *only* invoke expensive planning when the current situation is already “known.” A statistically “known” prediction means the agent knows with high probability that its prediction is almost as correct as that of the Bayes optimal predictor. Due to this approach, the computational demands are reduced compared to a regular planner, and further, the agent will know when to stop its planning efforts—when everything is “known.”

### 4.2. Conclusion

Goal-driven exploration is very common in the traditional RL setting. In the pure-exploration setting, self-generated goals are needed. The agent described here generates goals based on its confidence in its predictions about how the environment reacts to its actions. When a state-action outcome is statistically unknown, the environment setting where that experience can be sampled becomes a goal. The agent uses planning to manipulate the environment so that the goal is quickly reached. Without planning, only local, myopic exploration behavior can be achieved. The result is a sample-efficient, curiosity-driven, exploration behavior, which exhibits developmental stages, continual learning, and skill acquisition, in an intrinsically-motivated playful agent. Key characteristics of our proposed framework include: a mechanism of informed exploration (with no randomness involved), a clear distinction between direct and planned exploration (i.e., planning is done only when all local instances are statistically known), and a mathematically-solid way of deciding when to stop learning something and when to seek out something new to learn.

### Conflict of interest statement

The authors declare that the research was conducted in the absence of any commercial or financial relationships that could be construed as a potential conflict of interest.

## Footnotes

^1^For more information on subspace planning, see related work in relational RL by Lang and Toussaint (2009).

^2^A more general framework would consider the multiclass and regression cases, which we leave for future extension.

^3^Note that the terms *weight vector*, *linear hypothesis*, *classifier*, and *learner* are fairly interchangeable for the purposes of this article.

^4^To allow generalization in learning, each weight vector is extended with one extra bias component, corresponding to an extra augmented feature of 1. Thus, |**u**| = |**w**| = |**x**_*t*_| = 22 in the implementation of the simulated environment, and |**w**| = |**x**_*t*_| = 7 in the implementation on the real robot.
